# Freshman Experiences Among Neurodivergent Students Following a STEM-Focused High School-to-College Transition Program

**DOI:** 10.3390/bs16010160

**Published:** 2026-01-22

**Authors:** Bryan K. Dallas, Shupei Yuan, Briona Humphrey

**Affiliations:** 1 School of Interdisciplinary Health Professions, Northern Illinois University, 1425 West Lincoln Highway, DeKalb, IL 60115-2828, USA; 2 Department of Communication, Northern Illinois University, 1425 West Lincoln Highway, DeKalb, IL 60115-2828, USA; syuan@niu.edu; 3 Department of Educational Technology, Research and Assessment, Northern Illinois University, 1425 West Lincoln Highway, DeKalb, IL 60115-2828, USA; z1862046@students.niu.edu

**Keywords:** neurodivergent students, STEM education, transition to college, interview, students with disabilities

## Abstract

Little research exists that focuses on the transition experiences of students with disabilities (SWDs) from high school to college and scholarly investigation of science, technology, engineering, and math (STEM) pathways for neurodivergent students is emergent. The purpose of this current study is to better understand the experiences and perspectives of college freshman with disabilities, following participation in a STEM-focused high school-to-college transition program. Participants in this study completed a yearlong STEM-based college transition program in 2023, followed by a follow up survey and semi-structured interview during their freshman year in college. Results outline participant successes and challenges related to multiple college and career readiness factors. Most participants experienced a successful transition to their first semester in college, continued engagement in STEM-related career development, and several social and extracurricular activities. Future practice and research recommendations are provided.

## 1. Introduction

In the United States (U.S.), many people with disabilities (PWDs) experience fewer educational and employment opportunities compared to the general population (U.S. Bureau of Labor Statistics, 2025). The National Center for Education Statistics reported that only 40.4% of undergraduate students with disabilities (SWDs) attained their degree compared to 50.6% of their peers without disabilities (Hinz et al., 2017). Additionally, PWDs are much less likely to work in management, professional, and related occupations, which include Science, Technology, Engineering, and Math (STEM) fields, than their nondisabled peers (37.9% vs. 44.1%, respectively) (U.S. Bureau of Labor Statistics, 2025).

Pursuing college continues to be one of the best ways for SWDs to enter the workforce, increasing their employment rates overall (U.S. Bureau of Labor Statistics, 2025). Additionally, STEM occupations are growing in the U.S. and PWDs, including those who are neurodivergent, can help fill available jobs (Griffiths et al., 2021). However, the U.S. Department of Health and Human Services (2017) has highlighted the need for targeted interventions to improve postsecondary outcomes for neurodivergent students, including those with Autism Spectrum Disorder (ASD). In addition to ASD, neurodivergent groups can include those with several neurodevelopmental conditions (e.g., learning disabilities (LD), attention deficit hyperactivity disorder [ADHD]) and various mental health diagnoses (e.g., anxiety disorder, depression, obsessive–compulsive disorder).

The transition from high school to college can present significant challenges for SWDs, including neurodivergent students. For example, while students with ASD can exhibit strong academic abilities, especially in STEM disciplines, they often encounter barriers related to social communication, executive functioning, and self-advocacy skills (Falvo & Holland, 2018; Grandin & Duffy, 2008; Wei et al., 2013). These challenges may contribute to lower college enrollment and completion rates for individuals with ASD compared to their neurotypical peers (Taylor & Mailick, 2014). McLeod et al. (2019, 2021) found that college students with ASD self-reported more negative outcomes related to academic performance, social relationships and bullying, and physical and mental health compared to neurotypical students.

The literature on the transition to college among SWDs is varied and includes research that focuses on a specific population (e.g., only participants with ADHD), as well as more heterogenous groups. Even so, there are some common themes that can be found across research studies that can be applied to multiple populations and settings. In a qualitative study on the college decision-making process of a heterogenous group of 20 high school SWDs, Petcu et al. (2025) recommended that high schools begin working on college and career readiness (CCR) activities (e.g., goal setting, decision making, and self-advocacy) with students by the ninth grade. Participants reported not fully participating in all college-related activities offered (e.g., college fair) by their high schools. The authors recommended that school counselors and special educators encourage consistent parental involvement (e.g., IEP meeting attendance) and emphasize the importance of student engagement in the college exploration process.

Neurodivergent students may also need these types of supports to develop college-related skills before leaving high school. In a large study comparing college student outcomes, students with ADHD experienced more academic difficulties during their first two years, compared to a general population of students (DuPaul et al., 2018). Students’ self-reported motivation to study during their first year in college was a significant predictor of their second-year grade point average (GPA). The authors highlighted the need for postsecondary institutions to support students (e.g., enhance study skills and increase academic motivation) early on in college to bolster their academic success. Although legislation such as the Individuals with Disabilities Education Improvement Act (IDEA, 2004) requires K-12 school districts to prepare SWDs for adult life, not all high schools provide a wide range of CCR opportunities (Mazzotti et al., 2024).

While the ASD population has been increasingly studied over the last few decades, there remains a significant gap in knowledge pertaining to their experiences and outcomes after high school. Little research exists that specifically focuses on the transition experiences of students with ASD from high school to college (Austermann et al., 2023; Madaus et al., 2022) and scholarly investigation of STEM pathways for students with ASD is emergent (Nachman et al., 2024). For example, Kouo et al. (2021) reported finding sparse literature and empirical studies on STEM-specific supports for students with ASD in engineering. Furthermore, a gap exists in knowledge on the experience and outcomes of neurodivergent students based on multiple demographically relevant factors (e.g., race, gender).

In their systematic review of the literature, Nachman et al. (2024) found that involvement in STEM programming improved ASD students’ developmental skills, but that participants were not demographically diverse. The authors recommended more research on individuals with ASD pursuing STEM employment, featuring more diverse participants while exploring their unique experiences and perspectives in education and employment. These findings align with previous research on SWDs pursuing STEM fields. For example, using data from the National Longitudinal Transition Study-2, A. Lee (2014) found that female SWDs were underrepresented in STEM majors in postsecondary institutions. In 2-year or community colleges, White and Asian-American students with disabilities substantially outnumbered other racial groups in STEM majors. These findings were similar to Lee’s previous research (A. Lee, 2011) on nondisabled students that showed underrepresentation of female students in STEM majors and overrepresentation of White and Asian-American students compared to other race/ethnic groups. A. Lee (2014) recommended that future studies analyze whether different effects of selected factors on STEM major choices exist by demographic characteristics, such as race/ethnicity backgrounds. To address underrepresentation of SWDs in college, some researchers have suggested the use of scaffolding supports to assist individuals as they move from secondary school settings to postsecondary institutions (Dunn et al., 2015; Author Hidden). Scaffolding supports are meant to help guide students toward independence and ideally are implemented before students leave the high school setting. Multiple stakeholder groups can provide these supports including parents (e.g., reinforce good hygiene, time-management, paying bills, laundry), high school counselors and teachers (e.g., practice self-advocacy skills), and colleges (e.g., tutor support, transition experiences). For example, Stevens et al. (2024) found that students with ADHD reported that adequate parental support was fundamental in their transition to college. Similarly, in another study on the transition experiences of SWDs (i.e., learning disability, blind, hearing impairment, attention deficit hyperactivity disorder), participants reported benefit from informal transition supports such as family support and self-advocacy skills (McCall, 2015).

There is some evidence that college transition programs may be beneficial for neurodivergent students. Dwyer et al. (2023) recommended these types of programs for neurodivergent students (i.e., ASD, ADHD) as they matriculate to college and again as they leave to pursue employment. DuPaul et al. (2021) also recommended implementing support services for students with ADHD before starting college, specifically focusing on enhancing executive functioning skills and managing any co-occurring depressive symptoms. In their study on parents of college students with ASD, Madaus et al. (2022) reported that early transition supports and opportunities related to instruction, extracurricular enrichment activities, and practicing executive functioning and independent living skills may enhance successful transition to college. Reis et al. (2022) identified several characteristics among academically successful college students with ASD including attending residential summer programs as well as interest-based extra-curricular activities, advanced educational experiences, and other enrichment opportunities. The researchers recommended further investigation of the experiences of ASD students who both succeed and struggle in high school and college, to create research-based support programs and enhance services for this population. Likewise, Griffiths et al. (2021) explored the impact of a STEM school on SWDs and recommended that future research should explore the impact that STEM-specific programs have on students’ college and employment outcomes. Additionally, Dunn et al. (2015) found that collaboration among peers with ASD played a critical role in supporting the learning that occurred in a vocational exploration technology program for youth. Career exploration using interests in technology can provide opportunities for students to develop social and technical skills needed later for employment.

Throughout this manuscript, we use “students with disabilities (SWDs)” to align with the legal and educational categories used by schools, state agencies, and the transition program. Many of these students are also neurodivergent (e.g., ADHD, autism, learning disabilities). Our use of disability language is grounded in a social/interactional model, which emphasizes how educational environments and supports shape students’ opportunities, rather than framing neurodivergence as an individual deficit. We therefore use both terms intentionally: “SWDs” when referring to formal eligibility and services (e.g., DRC accommodations), and “neurodivergent students” when foregrounding diverse ways of thinking and learning.

There seems to be a critical need for targeted support programs that enable neurodivergent students to successfully transition to college and pursue STEM careers, thereby enhancing their employment opportunities and contributing to a more diverse and inclusive workforce. Based on the preponderance of evidence, best practice suggests that early exposure to college-level coursework, mentorship opportunities, and individualized support in areas such as executive functioning and social communication can significantly improve outcomes (Anderson et al., 2020, 2024).

### 1.1. STEM-Focused High School-to-College Transition Program

Participants in the current study previously participated in a STEM-focused college transition program in 2023, while they were still in high school. The program is supported by state grant funds and serves approximately 15 high school SWDs per year, as they prepare for college. The setting for this program is a public 4-year university, with a total student population of approximately 15,000 students. Criteria for admission to the program includes: (1) high school SWDs in their junior year; (2) documentation such as an individualized education plan (IEP) or 504 accommodation plan; (3) willingness to engage in STEM career exploration; and (4) intent to enroll in postsecondary education. SWDs were recruited for the transition program by contact with special education school districts throughout the state. To facilitate inclusion of students from various backgrounds, the program was completely free for participants, including reimbursements for family travel. Participant engagement methods during the yearlong program included e-mail, telephone, and Zoom videoconference calls, as well as multiple in-person visits to campus throughout the year. The Moodle online learning management system (LMS) was used to deploy program announcements, digital resources and information, program photos, and Essential Employability Skills (EES) learning modules (Illinois State University, 2025).

Program participants started the program during their junior year of high school with a needs assessment, career interest assessment, spring orientation, followed by a 5-day summer residential immersion program in July. The 5-day summer experience ran from Sunday evening through Friday afternoon and included (1) integrated residence halls and dining, (2) integrated recreation and socialization activities, (3) STEM exploration and related activities, and (4) an introduction to college academic support and accommodations. A significant portion of the program focuses on introducing participants to postsecondary STEM majors and careers, as well as interaction with university STEM faculty and staff. In the fall of their senior year in high school, participants are offered an opportunity to complete an EES online course, complete mock job interviews, participate in a university-wide STEM Fest event, and work with program staff to apply to the postsecondary institution of their choice. Once participants completed all program activities, applied to college, and completed the free application for federal student aid (FAFSA), they received a stipend to help pay for college equipment and supplies (e.g., laptop, backpack).

**Figure 1:**
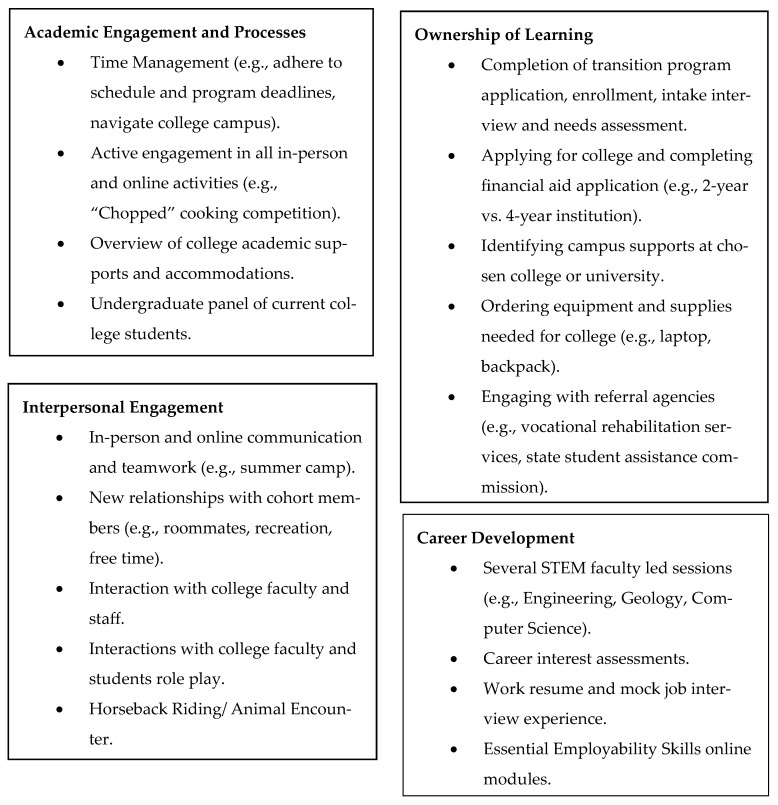
Transition Program Components and College and Career Readiness Domains.

See Figure 1 on how specific transition program components correspond with established CCR transition areas of Academic Engagement and Processes (AEP), Ownership of Learning (OL), Interpersonal Engagement (IE), and Career Development (CD) (A. R. Lombardi et al., 2023, 2025; Morningstar et al., 2017). AEP include strategies that students use to engage with academic content, OL includes academic and career growth by setting goals and implementing decisions, IE include the social skills needed to communicate with diverse stakeholders (e.g., students, faculty, staff) in academic settings, and CD includes activities that facilitate the successful transition to multiple settings including employment, postsecondary education, and surrounding community (A. R. Lombardi et al., 2023, 2025).

### 1.2. Transition Program Assessment and the Current Study

Prior to the current study, the authors reviewed the grant program’s survey results. Program survey data show that participants were generally satisfied with the program, leaving the program more familiar with STEM career opportunities, more aware of college supports available for SWDs, and more confident in their ability to identify college accommodations and supports. However, it remains unclear how well students are prepared academically and specific challenges that they may encounter. The purpose of this current study is to better understand the experiences and perspectives of college freshman with disabilities, following participation in a STEM-focused high school-to-college transition program. Research questions that helped guide the study were developed and based on research on college and career readiness (CCR) (A. R. Lombardi et al., 2023; Morningstar et al., 2017) and included:

RQ1. How much academic and extracurricular engagement did students achieve during their freshman year in college?

RQ2. What were the experiences and perceptions of students during their freshman year in college?

RQ3. How do students decide to pursue one or more areas of study in college?

RQ4. How well did the college transition program help prepare students for college?

## 2. Materials and Methods

### 2.1. Recruitment

The researchers gained approval to conduct the current study through the university’s institutional review board, and recruitment materials emphasized that participation was voluntary, would not affect students’ access to services, and that responses would be de-identified before analysis. A total of twenty high school SWDs were served in the 2023 transition program cohort. Two of those students opted to wait to attend college for various reasons and plan to attend in the future. An additional student opted to work after high school and reported no immediate plans to attend college. Therefore 17 students entered college in the Fall of 2024 and were recruited for the current study. Students were contacted via university email with an initial invitation and one reminder message. Twelve of those students responded and completed the survey. Ten students completed a subsequent interview for a 59% response rate. Participants received a $200 check after completing both the survey and interview. Data was collected from participants at the end of their first semester in college. An electronic survey and subsequent interview were used to collect data. Demographic information gathered from the electronic survey among the 12 participants can be found in Table 1.

### 2.2. Instruments

Instruments utilized for this study included an electronic survey and semi-structured interview. Instruments were developed by the authors in consultation with the University’s research methodology services and based on a review of the literature. The electronic survey included items related to the type of institution attended, course-load, campus supports used, living arrangements, work/volunteer hours, and evidence of advanced placement (AP) or dual credit courses prior to college.

### 2.3. College and Career Readiness

The semi-structured interview questions were based on a combination of study research questions and college and career readiness (CCR) transition related areas of Academic Engagement and Processes (AEP), Ownership of Learning (OL), Interpersonal Engagement (IE), and Career Development (CD) (A. R. Lombardi et al., 2023, 2025; Morningstar et al., 2017). Additional interview questions were related to the impact of the yearlong transition program. Example survey items are listed in the Appendix A. Coding of the qualitative data included using theoretical thematic analysis (Braun & Clarke, 2006) based on A. R. Lombardi et al. (2023, 2025) four CCR domains of Academic Engagement and Processes (AEP), Ownership of Learning (OL), Interpersonal Engagement (IE), and Career Development (CD). Three members of the research team served as primary coders. We developed an initial codebook deductively from the CCR domains and then inductively refined subcodes to capture unanticipated, recurring patterns in the data. Both coders independently coded two transcripts and compared coding to discuss discrepancies, clarify code definitions, and revise the codebook. Remaining transcripts were then divided between coders, with regular meetings to review challenging excerpts and reach consensus. Disagreements were resolved through discussion until consensus was reached, and a senior member of the team was consulted when needed. We maintained an audit trail documenting codebook revisions and analytic decisions throughout the process.

## 3. Results

Table 2 provides descriptive statistics from the electronic survey completed at the end of participants’ first semester in college. 100% (N = 12) of respondents attended college full-time and only one (8.3%) student reported living alone. Participants reported taking a range of 3–6 courses (M = 4.25) and 12–18 credit hours (M = 14.5). Eleven participants (91.7%) indicated that they expected to attend college the following semester.

### 3.1. Academic Engagement and Processes and Interpersonal Engagement

RQ1 explored the level of academic and extracurricular engagement during participants’ freshman year of college and addressed the CCR domains of AEP and IE. Overall, participants shared their experiences engaging with general campus facilities and support units such as the disability resource center (DRC), as well as their classroom engagement and extracurricular activities. Participants used a variety of resources on campus, including programs specifically designed for the first year, or campus facilities or software.

“...they had this website called glean, which is like a recording site and a site that can organize my notes basically. It records, but so then I can listen back to lectures, and it allows me to take notes live. I missed something in the lecture that was spoken I can get everything I can have written down, but then also have the spoken notes so then I can make notes from that.”

(Student #1, ADHD and Anxiety)

Most students shared that they received information about campus resources during the campus visit orientation and had positive experiences engaging with them.

“The school has a lot of posters, and they send out messages on your phone. And so usually, like at the beginning of the year, they sent like three messages about the bus system and how it worked. During the first welcome week there was a bunch of like advertising for the rec center and stuff and then I heard about the doctor’s office and stuff through friends.”

(Student #2, ASD)

Likewise, another student shared their experience taking a course that included coverage of campus supports:

“As freshman in college, we were put into this class called First Year Success. And my professor told us about all the things that you can go to, if you need help. He talked about getting a U-Pass if you’re a commuter student and you don’t want to drive here. Which is very helpful and very useful. I used the commuter train and the bus system.”

(Student #3, LD)

Participants also shared their positive experience with campus resources:

“But I also like to go to the writing center which was a lot of help. A writing major that they had would also give me feedback of what to say and, you know, to take this out or put this in and they would just give me their opinion, and they would just you know correct me on some of my errors.”

(Student #3, LD)

Interestingly, only 33% of survey respondents indicated using their campus DRC. This generally indicated that most participants were not requesting formal academic accommodations from their instructors and not sharing this information with classmates (e.g., group work). Students shared the reasons they did not use DRC support, such as missing the deadline to submit:

“No, I was going to, but I think I missed the deadline. But I was just going to get extra time on the homework and stuff. I did all right, but I still, there’s like a lot of homework that I just didn’t do or get to.”

(Student #2, ASD)

Or that they just did not feel the need:

“I feel like I go to those things, if I really need them, I’ll get them if I am really struggling, but if I can figure it out for the most part, then I don’t. If I need like a little bit of help also and I just can’t figure out whatever. Then I’ll go to them. But nine times out of 10, if I can figure out myself and I can get it right, then I say don’t really go to them.”

(Student #4, ADHD)

“I mean, the main reason was because I wanted to kind of test myself and kind of have that independence, if that makes sense. When it came to deadlines, I always made sure my stuff was turned in on time. Instead of asking for a later due date.”

(Student #3, LD)

A few participants did use DRC services and shared the usefulness they found from such a resource:

“They’ve helped me like get, like accommodations for my classes and a living space that’s close to the quad that allows me to get to class on time.”

(Student #5, ADHD and Executive Functioning/Processing Speed Disorder)

Multiple students mentioned accommodations like extra time needed with course work:

“I’d say they helped me with time in classes. Working or completing assignments, I guess. I do get pretty slow when I take tests and stuff like that. So, I will need extra time.”

(Student #6, LD, ADHD, and Speech/Language Impairment)

“I use extended time on tests, no judgment and things, but most of the teachers just put a big fricking timer on the wall which stresses me the hell out because if I can see a timer, my immediate reaction is frick, frick, frick, get it done....”

(Student #7, ASD and Anxiety)

In some situations, students recognized that they needed DRC support at later stages of the semester:

“I’d say I managed poorly during these times without any accommodations, and I feel like I should have done this earlier in the beginning.”

(Student #6, LD, ADHD, and Speech/Language Impairment)

Additionally, students also discussed their interpersonal engagement with faculty and peers during the first semester. Generally, most students shared positive experiences in terms of sense of belonging. They acknowledged the academic challenges while being able to seek support from faculty:

“In the beginning months, August through September it was kind of challenging because there is some stuff that my professors were talking about that I didn’t really understand, especially the classwork that I don’t think I was fully prepared for. But that is something that I had a one-on-one conversation with my professors. They were truly understanding, and they helped break it down for me a lot more when it came to the topic.”

(Student #3, LD)

“My chemistry teacher was really accommodating. He said that I could take the test at the testing center or in the department.”

(Student #8, ADHD and Depression)

Multiple students mentioned that they used tutors or engaged in study groups.

“I just started using teaching assistant (TA) support because I needed to get out of my room like I would just be trapped in my room for like hours in the day. I don’t think I started seeing my TAs until like later in the semester, so I feel like that helped a bit.”

(Student #5, ADHD and Executive Functioning/Processing Speed Disorder)

However, the frequency of academic supports varied, with some using them “two to three times a month” (Student #8, ADHD and Depression) and others only use a few times per semester.

Moreover, almost all participants mentioned one or two extracurricular activities that they were involved in. One student discussed why they continued in the marching band in college:

“I was in marching bands all throughout middle school and high school, and I just really loved it” (Student #5, ADHD and Executive Functioning/Processing Speed Disorder). Other students shared their extracurricular activities such as participating in sports (e.g., track), Information Technology (IT) academy or pre-vet club. Multiple participants also mentioned that they enjoyed music or playing video games with friends during their free time.

### 3.2. Ownership of Learning

RQ2 explored the experiences and perceptions of students during their freshman year in college and addressed the CCR domain of OL. One salient finding is that many participants did not realize the big difference between high school and college, and this difference is reflected in many perspectives. Course styles are one of the main differences:

“Compared to high school, the amount of classes felt a lot different in the credit system.”

(Student #6, LD, ADHD, and Speech/Language Impairment)

“I didn’t completely expect the amount of homework and how I thought it was going to be.”

(Student #2, ASD)

“The level of preparation I didn’t really grasp going from high school to college because there’s some stuff that they don’t tell you in high school that you need in college.”

(Student #3, LD)

Another main difference comes from self-time management:

“I wasn’t prepared for the time management that would be required.”

(Student #7, ASD and Anxiety)

“I would say the parts of the transition that I was least prepared for or that were hard, kind of like I said, like getting used to having that freedom...That was definitely a little different because, you know, in high school your teachers are always reminding you like, oh, this assignment’s upcoming or this is due.”

(Student #9, Physical Disability)

Because of these changes, students reported having up and down moments within the semester. Students shared mixed responses to their feelings of the first semester: challenges are mentioned as well as confidence and enjoyment.

“My confidence really fluctuated throughout. In the beginning, I was pretty confident. And then as I went on, I was getting a bit less confident then when finals were coming, I knew I was prepared going into the tests. And then I was getting more confident again. I’m a little surprised, I guess, by how much better I’m enjoying college than I thought.”

(Student #8, ADHD and Depression)

“Now I feel… confident that I will be able to pass all of the classes that I have currently taken because I have a much better support system now and I feel that the courses that I’ve taken are manageable to me.”

(Student #7, ASD and Anxiety)

All the participants who took AP or dual credit courses during high school reported success in their first college semester. For those students, the transition seemed to be less stressful:

“I did take a lot of honors or AP courses throughout my years in high school, but I know like going prior to getting to college, a lot of my teachers in high school would say like, oh the workload in college is much more intense. And so I kind of went in expecting like, not that it wasn’t intense, but it was a lot more manageable than I thought it was.”

(Student #9, Physical Disability)

### 3.3. Career Development

RQ3 explored CD and how students were making decisions related to their focus of study in college. Most participants reported not working or volunteering during their first semester. Therefore, most participants were addressing CD through academic career exploration. The yearlong transition program focused on STEM-related career exploration, and most participants reported pursuing STEM majors and careers:

“Yeah, I’m doing…Computer science and then I think I’m double majoring. I’m still talking it out with my…my advisor, but I think I’m double majoring in math statistics.”

(Student #2, ASD)

“I have narrowed it down to like two or three. So I either want to do mechanical engineering maybe aerospace engineering. Those are very close, like either something like that or theoretical physics, astrophysics, that kind of thing. And there are some career paths where there’s a lot of overlap in the research fields especially so I don’t need to choose right away.”

(Student #8, ADHD and Depression)

“So yeah, so my major is animal sciences and I know like some schools do it differently, here it’s just like kind of like the regular one you pick your major as you apply your or like when you apply and then when you’re accepted and that’s your major. I’m still working or figuring out now. It’s kind of early, but I might end up later on picking up a chemistry minor because my major requires five chemistry courses. And to get the chemistry minor, it’s like one or two extra classes.”

(Student #9, Physical Disability)

Importantly, participants shared insight on what led them to pursue specific fields in college. In general, personal interests developed early on, along with experiences gained through activities and programs they participated in before college, and contributed significantly to their choice of academic focus:

“So, it was my first semester of my senior year. I took one of those classes [Computer Science] because it was just a random elective that filled in with my schedule and....I feel like I was really good at it and I was really like kind of, I want to say like passion about it. But it was a skill that just clicked with me. I mean, I took. I took a little bit of courses in like middle school so that also kind of helped, but I felt like that was the right thing for me to do.

(Student #4, ADHD)

“I did an internship where I got to see what being an engineer is like. And things like that have helped me explore what various career paths and what the education everything is like. And I think that’s helped me to see what things I like about each career and major and what I probably prefer not having to do. And we also had something in my engineering class last semester where every other week there would be two guest speakers who would come in and talk about their type of engineering. I guess that also helped. I guess I watch a lot of like physics and engineering videos on YouTube that probably also builds into my career exploration. I’m also in a program called Engineering Pathways where you apply to a specific university and then you do two years at a community college where you meet the requirements like GPA and other stuff.”

(Student #8, ADHD and Depression)

“I guess Boy Scouting is a big part of it. I’ve always been very interested in the environment and I love nature a lot. And I just want to protect it. Every day like we used to do like roadside cleanup in Boy Scouts for like community service hours, which was a requirement Every time I did that, I was just utterly disgusted of how Awful people could be just throwing trash every which way, not caring at all and I just made me want to like fix it.”

(Student #7, ASD and Anxiety)

RQ4 focused on how the transition program during their senior year of high school helped them prepare for college. One feature of the transition program that participants highlighted was the experience navigating a university campus and living in the residence halls for a week in the summer before their senior year in high school. To a certain degree, this experience provided students with opportunities to acclimate to the college environment, practice independent living and support routines, and develop a clearer sense of what college life would actually be like, as reflected in the following comments:

“It helped me like on the environment side of things, you know it was very different from what I was used to. It kind of gave me a taste of what college life is going to be like. So I feel like that’s what really helped. I feel like the most helpful was. One staying in a dormitory with another person to exploring the campus and like knowing everything that’s around it and like how to utilize it, I feel like that’s what helped me the most.”

(Student #4, ADHD)

“I worked with personal care attendants (PCAs) for the first time. I think that helped tremendously. When I got to my university, we have an accessible dorm. And then we have PCAs who will come in the morning and night or throughout the day to help us. And for some people I know here, this was their first time working with PAs. And I know for a lot of people, it’s a little hard because usually their parents or family members would help them, and I think that part of the transition program helped me a lot.”

(Student #9, Physical Disability)

“It felt like how being in college would feel for me, actually living there, seeing other people, how the buildings are going to be, and how long is it going to take for you to actually get to class because the campus is big. I feel like it helped me along the way, that I’m more prepared and more serious.”

(Student #10, LD and Speech/Language Impairment)

Additionally, participants highlighted their experiences socializing and collaborating with both their peers and faculty:

“And it also helped me learn how to be social with people you just met. I got to be more social during the program.”

(Student #1, ADHD and Anxiety)

“There’s times that we had to work in groups, really working together as a team, and there were some teamwork skills that we did. Communication skills was another big one.”

(Student #3, LD)

“A big thing would be a big thing would be career exploration with engineers. I remember I got to speak with an engineering professor.”

(Student #8, ADHD and Depression)

In some cases the program helped alleviate participants’ anxiety about college:

“I feel like the biggest thing, at least immediately was it just got me thinking about college in like a good way and I was a lot more excited, I guess. And I guess a lot of the fears of certain aspects of it were lessened.”

(Student #8, ADHD and Depression)

“The program helped because it got me used to college too, like college life because I was worried about that. I was worried about what is was going to be like living on campus and stuff.”

(Student #1, ADHD and Anxiety)

Results of the semi-structured interviews found that 8 out of 10 interviewed participants continued to their second semester in college. One of these participants (Student #7, ASD and Anxiety) attempted to live on campus during their first semester, however decided to return home early in the semester with a switch to only online classes. Two participants reported leaving college after their first semester. One of these students (Student #10, LD and Speech/Language Impairment) was attending community college, living at home, and working evenings while trying to attend school during the day. An additional participant (Student #6, LD, ADHD, and Speech/Language Impairment) who left college after their first semester was living on campus and was enrolled in 6 courses (i.e., 17 credit hours). The two participants who did not continue in their second semester reported using little to no campus supports (e.g., DRC) and did not complete AP or dual credit classes during high school. These participants reported plans to seek help from state division of vocational rehabilitation services, look for work in the short-term, and will consider returning to college in the future.

## 4. Discussion

This study examined the experiences and perceptions of college SWDs, following participation in a STEM-focused high school-to-college transition program. Our study is in response to past research recommending more exploration of STEM pathways among neurodivergent students (e.g., ASD) and their experiences moving from high school to college (Austermann et al., 2023; Kouo et al., 2021; Madaus et al., 2022; Nachman et al., 2024; U.S. Department of Health and Human Services, 2017). Using the CCR framework, we organized students’ narratives around Academic Engagement and Processes (AEP), Ownership of Learning (OL), Interpersonal Engagement (IE), and Career Development (CD) to better understand how the transition program supported key college- and career-readiness competencies. Results of the current study align with results from Reis et al. (2022) who highlighted characteristics among academically successful college SWDs. Participants in the current study attended a residential summer program and most participants had a successful first semester in college. Based on participants responses, involvement in the residential summer program was the most helpful in preparing them for college. This makes sense as it was the most intense part of the program, requiring participants to live on campus for a week and consistently engage in CCR activities. Other components of the program were online and could be completed based on individual participants’ schedules. Multiple successful participants in the current study participated in interest-based extra-curricular activities (e.g., band, track), advanced educational experiences (e.g., advanced placement/dual credit courses), and other enrichment opportunities (e.g., transition program, campus supports, Engineering Pathways). Previous research on predictors of positive outcomes (e.g., retention, graduation) in college includes academic preparation (e.g., high school grade point average) (Yu et al., 2018), level of self-advocacy skills and executive functioning (DuPaul et al., 2018; Y. Lee et al., 2012), and use of campus and social supports (e.g., DRC, inclusive campus) (Blasey et al., 2023; Los Santos et al., 2019; Maslow et al., 2012; Pingry O’Neill et al., 2012; Troiano et al., 2010). The current study adds to this literature by highlighting the experiences and outcomes of new students in their first semester, following a yearlong college transition program. Participants’ long-term success in college might be improved if more participants choose to use campus supports (e.g., DRC) in future semesters. Use of campus supports might also help participants continue to develop their self-advocacy and executive functioning skills.

The two participants in the current study who did not immediately continue in their second semester in college did not report engaging in all the success factors that Reis et al. (2022) outlined in their study and engaged in practices that might have negatively impacted on their academic performance (i.e., employed in the evenings, enrolled in 6 classes). Although each student’s situation and experience are unique, it might be helpful for college transition programs to address how students might structure a school–work–life balance before starting college. For example, some students might be more successful going to college part-time, or full-time but not exceeding 4 classes (e.g., 12 credit hours) during one semester.

Despite having an IEP or 504 Accommodation plan in high school, most students in the current study reported not using campus supports, including disability-related resources like a DRC. Reasons varied on why participants chose not to use their campus DRC, however previous research has highlighted some of the reasons for nondisclosure that may include a desire to avoid stigma or negative social reactions (e.g., professors, classmates), lack of adequate medical documentation, lack of knowledge or training on disclosure and accommodations, as well as lack of quality DRC services or accommodations that were not useful (Marshak et al., 2010; Toutain, 2019). Interestingly, one participant reported that they missed the deadline to register for academic accommodations. Colleges and universities do not place time limits on when a student can disclose their disability and request academic accommodations. Therefore, this participant might have been misinformed or hesitant to disclose the reason(s) why they did not contact the campus DRC. College SWDs who choose not to register with a DRC are less likely to have conversations with their course instructors and/or classmates about their learning needs and less likely to request and use academic accommodations. Several of the current study participants indicated that they would consider using campus supports in future semesters.

Additionally, frequency of use of campus supports varied among participants. Although study participants were informed about campus resources during the yearlong transition program, they did not necessarily make use of these supports after enrolling in college. From a CCR perspective, this suggests that OL and IE competencies related to self-advocacy, disclosure, and resource navigation were only partially developed, indicating a need for more explicit, practice-based preparation in these areas. Previous research on self-advocacy among SWDs suggests that education on postsecondary policies and procedures, coupled with practical or role-playing experience may lead to an increase in use of campus resources (e.g., academic accommodations) (Holzberg & Ferraro, 2021; Pfeifer et al., 2021). It may be helpful for future college transition programs to emphasize action-oriented “how-to” steps that encourage students to set up services early in their semester so that they can be accessed quickly when needed.

Reis et al. (2022) recommended further investigation of the experiences of students who both succeed and struggle in high school and college to create research-based support programs and enhance services for this population. Based on the current study results and previous literature, we can make recommendations to improve college transition programs for new SWDs. For example, it may be helpful to screen participants earlier before their senior year in high school regarding involvement in extra-curricular activities, AP/dual credit course completion, and use of high school academic supports. Given the growing demand for STEM professionals and the underutilization of talent among neurodivergent individuals, research into effective transition programs is both timely and essential. Additionally, future research should specifically examine how transition programs can be designed to increase access to and persistence in STEM majors and careers for SWDs, for example by integrating STEM-focused mentoring, early exposure to college-level math and science coursework, supported experiences in laboratory or research settings, and collaboration with disability resource offices to address access barriers in STEM learning environments. By addressing the unique challenges faced by this population and leveraging their strengths, such programs can serve as a vital bridge between secondary education and meaningful participation in STEM higher education and careers. Our findings suggest that STEM-focused exploration activities within the transition program contributed to CD competencies by helping some students confirm their interest and sense of belonging in STEM, while enabling others to make more informed decisions to pursue non-STEM majors. Future iterations of the transition program could be improved and expanded by starting earlier in high school, incorporating more sustained STEM-focused experiences (e.g., campus labs, mentoring, job shadowing), and strengthening collaboration among schools, families, and university disability/resource offices. These program enhancements should be systematically evaluated in future research to identify which components most effectively support SWDs’ access to and persistence in STEM pathways. Failure to address the transition needs of high school SWDs may lead to more negative outcomes such as lower quality of life among these individuals as well as continued unemployment/underemployment and greater dependence on taxpayer-funded social support programs.

### Limitations and Future Research

Study limitations include a small sample size, with twelve participants completing the initial survey and ten participating in the interview. Additionally, participants were all residents of the same midwestern state and these findings should be understood as context-specific and may not fully capture the experiences of SWDs in other regions or institutional settings. The study was a snapshot of outcomes after one semester and measuring additional long-term outcomes (e.g., retention, graduation) among this group would be ideal in order make a broader comparison to previous research. Even though this study focused on neurodivergent students, one participant had a primary physical disability and no other diagnosed impairment. Although confidentiality was maintained throughout the research, participants were not anonymous and therefore, in some cases, participants may have provided perceived socially desired responses to some of the survey and interview items (e.g., impact of college transition program). Despite the 2023 college transition program being free and open to inclusion of SWDs from various backgrounds, the demographic makeup of current study participants was similar to findings from A. Lee (2014) and Nachman et al. (2024) who found that most study participants were not demographically diverse based on factors such as race and gender. Half of the current study participants identified as White and over ninety percent identified as male. Experiences from a broader range of participants might have yielded different results. The 2023 transition program was open for anyone with an IEP or 504 accommodation plan to apply. To reach more demographically diverse students, it might be beneficial for future college transition programs to focus on serving specific subpopulations of SWDs (e.g., women with ASD).

Researchers have recommended further investigation of the college experiences and outcomes of SWDs to create research-based support programs and enhance services for this population. We echo this recommendation and encourage follow up studies with individual cohorts from college transition programs to measure college and employment outcomes. Additionally, findings from the current study can be used to improve transition programs for future students. For example, one student took six classes during their first semester in college and did not move on to the second semester of their freshman year. Another worked evenings and took classes during the day. SWDs can be counseled on the risks and benefits of employment while in college as well as manageable course load sizes. Finally, in their systematic review of assessment instruments designed for multiple college stakeholders including SWDs, A. Lombardi et al. (2018) found that most studies did not include rigorous research methods and recommended that future research address these gaps with rigorous study designs that use established instruments. Future studies on CCR could include using a larger number of participants along with standardized instruments like the College and Career Readiness for Transition measure (CCR4T) (A. R. Lombardi et al., 2023, 2025), as well as the addition of a control group of participants who did not participate in a yearlong college transition program while still in high school.

## Figures and Tables

**Table 1:** Demographic Information (N = 12).

Characteristics	Category	Frequency	Percentage
Gender	Male	11	91.6
	Non-binary	1	8.4
Primary Disability or Health Condition	Autism	4	33.3
	ADHD	4	33.3
	Learning Disability	3	25
	Physical Impairment	1	8.4
Race/Ethnicity	White	6	50
	Black	3	25
	Hispanic/Latino	2	16.6
	Asian	1	8.4

Note: Secondary health conditions among participants included anxiety, speech or language impairment, asthma, ADHD, hearing impairment, depression.

**Table 2:** End of First Semester Survey Results (N = 12).

Characteristics	Category	Frequency	Percentage
Postsecondary Institution	4-Year University	8	66.7
	Community College	4	33.3
Living Arrangements	On-Campus Residence Hall	7	58.3
	Family Residence	5	41.7
Campus Resources Used	Writing Center	4	33.3
	Tutoring	4	33.3
	Recreation Center	4	33.3
	Public Transportation	4	33.3
	Disability Resource Center	4	33.3
	Health/Medical Clinic	3	25
	Counseling Center	2	16.7
Paid Work/Volunteer Hours	None	9	75
	11–20 h per week	1	8.3
	21–30 h per week	1	8.3
	30+ h per week	1	8.3
College Credit in High School (e.g., advanced placement, dual credit)	Yes	8	66.7
	No/Unsure	4	33.3

## Data Availability

The original contributions presented in this study are included in the article. Further inquiries can be directed to the corresponding authors.

## Appendix A

Semi-Structured Sample Interview Questions

(1) Please describe how your transition from high school to college has gone. For example, what challenges have you experienced? What successes have you had?
(2) Overall, how prepared did you feel for college this semester?
  - In what areas did you feel most prepared?
  - Least prepared?
  - How confident do you feel in your ability to succeed in your college coursework and why?
(3) Have you decided on a college major and/or minor? Or, what subjects are you interested in?
  - How did you make this decision?
(4) Now let’s talk a bit about your college life. Did you engage in any extracurricular activities? If so, please describe.
(5) What other activities do you currently do for fun, leisure, or recreation?
(6) What strategies do you use to manage your time and stay organized in your studies?
(7) In the survey you mentioned that you used xxxx campus service.
  - How did you learn about these services?
  - Please explain how they assisted you.
(8) Did you disclose a health condition or disability to your college and/or use the campus disability resource center?
  - If so, please explain how they assisted you, including any successes or challenges experienced.
  - If no, is there any particular reason why you chose not to disclose?
(9) How do you talk with peers and professors regarding accommodations or your disability?
(10) In what ways, if at all, did the college transition program help prepare you for college?
